# Supplementary material for: OpenMM 7: Rapid development of high performance algorithms for molecular dynamics
Source: PLoS Comput Biol. 2017 Jul 26;13(7):e1005659. doi: 10.1371/journal.pcbi.1005659 (PMC5549999; doi:10.1371/journal.pcbi.1005659)
Supplement: S1 Source Code — (ZIP) [file pcbi.1005659.s002.zip › OpenMM-7.0.1-Source/docs/api-python/search.html]

Search — OpenMM 7.0.1.dev-5e86c4f documentation


# Search

Please activate JavaScript to enable the search
functionality.

From here you can search these documents. Enter your search
words into the box below and click "search". Note that the search
function will automatically search for all of the words. Pages
containing fewer words won't appear in the result list.

High performance molecular simulation on GPUs


### Navigation

- Application Layer
- Library Layer

©2015, Stanford University and the Authors.
|
Powered by Sphinx 1.3.1
& Alabaster 0.7.7
